# Long-term CT and US imaging of canine prostatic carcinoma after prostatic artery embolization: a 900-day follow-up case report

**DOI:** 10.3389/fvets.2026.1947473

**Published:** 2026-09-28

**Authors:** Saneyuki Isshiki, Sachiyo Tsuji-Kawahara, Mayu Ogata, Shinya Hisamoto, Moe Hashimoto, Tetsuya Hasegawa, Ikuo Tsunoda

**Affiliations:** 1 Kakogawa Animal Hospital, Kakogawa, Hyogo, Japan; 2 Department of Immunology, Kindai University Faculty of Medicine, Osaka, Japan; 3 Department of Microbiology, Kindai University Faculty of Medicine, Osaka, Japan

**Keywords:** endovascular procedures, interventional oncology, ischemic change, microspheres, prostate cancer, prostate neoplasms

## Abstract

Canine prostatic carcinoma is a highly aggressive malignancy with a poor prognosis and limited long-term treatment options. Prostatic artery embolization (PAE) has emerged as a minimally invasive alternative, but long-term intraprostatic imaging changes after PAE in dogs remain poorly documented. We performed PAE on a 13-year-old neutered male Chihuahua with prostatic carcinoma. Designating PAE as Day 0, we monitored the prostate, bladder, and lungs with computed tomography (CT), ultrasonography (US), and thoracic radiography for approximately 900 days. Clinical signs remained well controlled; the dog survived until Day 918. CT showed intraprostatic hypoattenuating lesions (Day 33) that enlarged with gas accumulation (Day 173); US showed hypoechoic lesions (Day 271) that became scattered hyperechoic foci (Day 678). Following PAE, the prostatic parenchymal volume decreased by approximately 33% and remained stable until Day 481. No serious PAE-related complications were observed. These findings suggest that PAE induced sustained ischemic, cavitary, and chronic remodeling changes within the prostate, which may have contributed to the prolonged local disease control observed in this dog. To our knowledge, this represents the longest time-course imaging reported after PAE in a dog with prostatic carcinoma and illustrates the potential of PAE as a feasible, minimally invasive treatment option in general veterinary practice.

## 1 Introduction

Prostatic carcinoma is a highly aggressive malignant tumor that primarily affects middle-aged and older neutered male dogs. Although the precise etiology of canine prostatic carcinoma remains unknown (1), chronic inflammation and oxidative stress have been proposed to contribute to the pathogenesis of benign prostatic hyperplasia (BPH) in dogs (2). At the time of diagnosis, it is often accompanied by local invasion of the bladder and urethra, regional lymph nodes, and distant metastases to the lungs, bones, and liver; the median survival has been reported to be 1 to 2 months (3). Surgical resection, most commonly total prostatectomy, is a primary treatment option, although radiation and drug therapies have also been used (1, 4–7). Surgical therapy can be expected to prolong survival, but has been associated with serious complications, including urinary incontinence (4, 6). Radiation therapy has also been shown to be effective for local control and for improving clinical signs, although acute and late radiation-associated toxicoses have been reported (7). It requires advanced equipment and specialized technical skills, limiting the number of facilities capable of performing it (5, 7). Although drug therapy, including non-steroidal anti-inflammatory drugs (NSAIDs) such as piroxicam, meloxicam, and firocoxib, and cytotoxic or molecularly targeted agents such as carboplatin, mitoxantrone, and toceranib, is less invasive than the two methods above, it rarely provides long-term control of prostatic carcinoma (1, 6). Thus, an efficacious and minimally invasive treatment for canine prostatic carcinoma has been needed.

Recently, prostatic artery embolization (PAE) has garnered attention as a minimally invasive treatment for prostatic carcinoma; PAE selectively embolizes the prostatic arteries and blocks blood flow to the prostate tissue, inducing ischemic changes (8–10). PAE has been widely used in humans as a treatment for BPH, and its application for prostate cancer has also been reported (10, 11). In dogs, experimentally, PAE has been reported to be efficacious for BPH; imaging and histological analyses have shown ischemic changes in prostate tissue and a reduction in prostate volume (12–15). Clinically, however, PAE for canine BPH remains limited, although it has been shown to be technically safe and feasible in dogs (12, 13). There are only two reports on PAE for prostatic carcinoma (excluding experimental animal models). Although these reports demonstrated the feasibility of PAE in dogs, a reduction in prostate volume, and improvement in clinical signs, PAE for canine prostatic carcinoma remains a new treatment modality (16), since interventional oncology in the veterinary field is still considered in its early stages of development (17). Culp et al. (18) reported a significant reduction in prostate volume 30 days after PAE and improvements in clinical signs, including dysuria, tenesmus, and lethargy, in 20 canine cases of prostatic carcinoma; Tiffinger et al. (19) reported a median survival of 13 months following PAE in six dogs with prostatic carcinoma (Table 1). Since the median survival time for canine prostatic carcinoma was as short as 1 to 2 months if left untreated (3), these findings demonstrated the promising efficacy of PAE. However, there are no case reports with long-term follow-up of intraprostatic lesions after PAE, with imaging evidence of change.

**Table 1:** Canine prostatic artery embolization (PAE) reports.

References	Disease	Cases	Imaging evaluation		Histology	Findings
			Method	Follow-up period^*^		
Jeon et al. (12)	BPH	5	MRI	3 months	Yes	Prostatic cystic change, glandular atrophy, and fibrosis with residual glandular tissue.
Sun et al. (13)	BPH	10	MRI, US	1 month	Yes	Prostatic cavity formation with residual glandular tissue; prostatic volume reduction.
Lucas Cava et al. (14)	BPH	5	MRI, Angiography	1 month	Yes	Prostatic infarction and cavitary formation, 1 week after PAE. Prostatic cavity formation, glandular atrophy, and fibrosis; prostatic volume reduction, 1 month after PAE.
Lucas Cava et al. (24)	BPH	10	MRI	1 month	No	Prostatic infarction and cavity formation; prostatic volume reduction, which was correlated with infarction extent.
Culp et al. (18)	Prostate carcinoma	20	CT, US	1 month	No	Prostatic volume reduction with clinical improvement. Long-term survival impact was not evaluated.
Tiffinger et al. (19)	Prostate carcinoma	6	Not reported	—	No	Clinical improvement and prolonged survival after PAE were found to be comparable to or better than previously reported outcomes (3–18 months).

BPH, benign prostatic hyperplasia; CT, computed tomography; MRI, magnetic resonance imaging; PAE, prostatic artery embolization; US, ultrasonography. ^*^Follow-up period indicates the longest duration of imaging follow-up after PAE.

In this study, we performed PAE for a canine prostatic carcinoma case and examined the long-term computed tomography (CT) and ultrasound imaging changes and clinical course of the intraprostatic lesion following PAE. We monitored the treatment's long-term efficacy for 2.5 years. We believe that our current report provides long-term imaging evidence that may inform the future evaluation of PAE as a minimally invasive treatment option in general veterinary practice.

## 2 Case description

A 13-year-and-6-month-old neutered male Chihuahua weighing 7.76 kg was presented to Kakogawa Animal Hospital (Kakogawa, Hyogo, Japan) on the day the owner first noticed acute vomiting, anorexia, and polydipsia. No urinary or respiratory signs were noted at presentation, with reportedly normal urination and defecation. The dog had well-controlled diabetes mellitus diagnosed 4 years earlier, managed with twice-daily insulin, and had been castrated at 11 years of age concurrently with excision of a perianal gland adenoma. Blood examination at presentation revealed markedly elevated hepatobiliary enzyme activities (alanine aminotransferase, 608 IU/L; alkaline phosphatase, 187 IU/L), hyperbilirubinemia (total bilirubin, 0.97 mg/dl), hyperlactatemia (8.8 mmol/L), mild thrombocytopenia, and hyperglycemia (209 mg/dl). Abdominal ultrasonography (US; Aplio 400, CANON MEDICAL SYSTEMS, Tokyo) revealed a gallbladder lesion suggesting gallbladder rupture or acute cholecystitis. The dog was hospitalized and stabilized overnight with intravenous fluid therapy and maropitant. The following day, whole-body contrast-enhanced CT (BrightSpeed Elite SD, GE HealthCare Japan, Osaka) and cholecystectomy were performed under the same general anesthesia. On CT, we incidentally found a mildly enlarged prostate with heterogeneous enhancement after administration of the contrast agent (Figures 1A, B). Abdominal US showed heterogeneous echogenicity of the prostate parenchyma and cystic hypoechoic areas compared with a prostate ultrasound image taken 3 years and 10 months prior to diagnosis (Figures 2A, B). Using CT and thoracic radiography, we also found a solitary, oval-shaped mass in the left caudal lung lobe. In this report, the PAE date was designated as Day 0; the date of the above imaging was Day −62.

**Figure 1:**
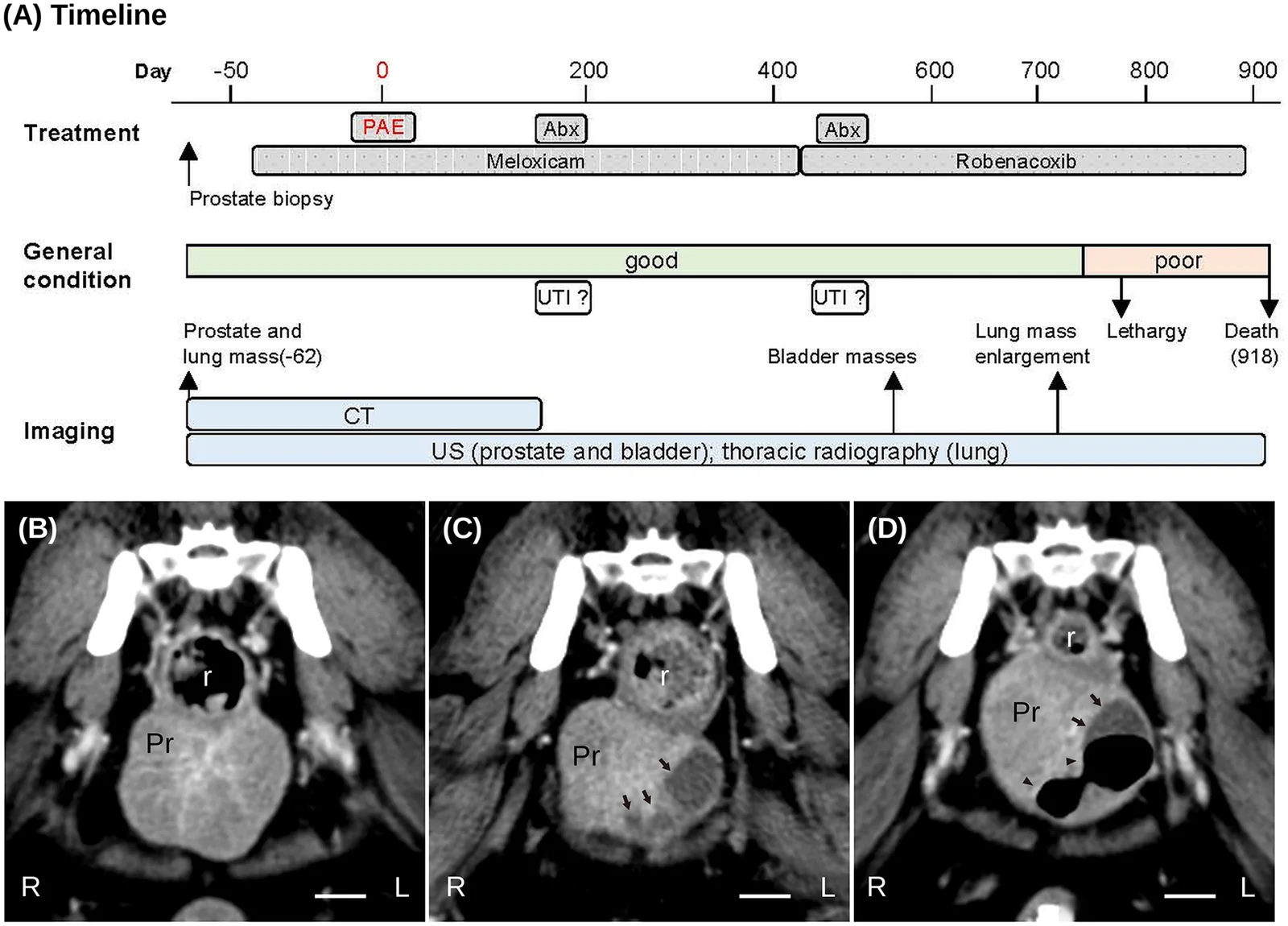
Case timeline and time-course prostatic imaging by contrast-enhanced CT. **(A)** Timeline of the case. Day 0 was defined as the day of prostatic artery embolization (PAE). Abx, antibiotics; US, ultrasonography; UTI, urinary tract infection. **(B)** On Day −62 (62 days before PAE), heterogeneous contrast enhancement was found in the prostate (Pr). r, rectum; R, right; L, left. **(C)** On Day 33 (33 days after PAE), hypoattenuating lesions (arrows) in the prostatic parenchyma were observed. **(D)** On Day 173, enlarged hypoattenuating lesions (arrows) with intralesional gas accumulation (arrowheads) were observed. Imaging parameters: **(B)** 120 kVp, 70 mA, and 3-mm slice thickness; **(C, D)** 120 kVp, 60 mA, and 3-mm thickness. Images are displayed using a soft-tissue window (window width, 320 HU; window level, 80 HU). Scale bar = 1 cm. CT images were reconstructed from the original acquisitions to display a comparable anatomic level.

**Figure 2:**
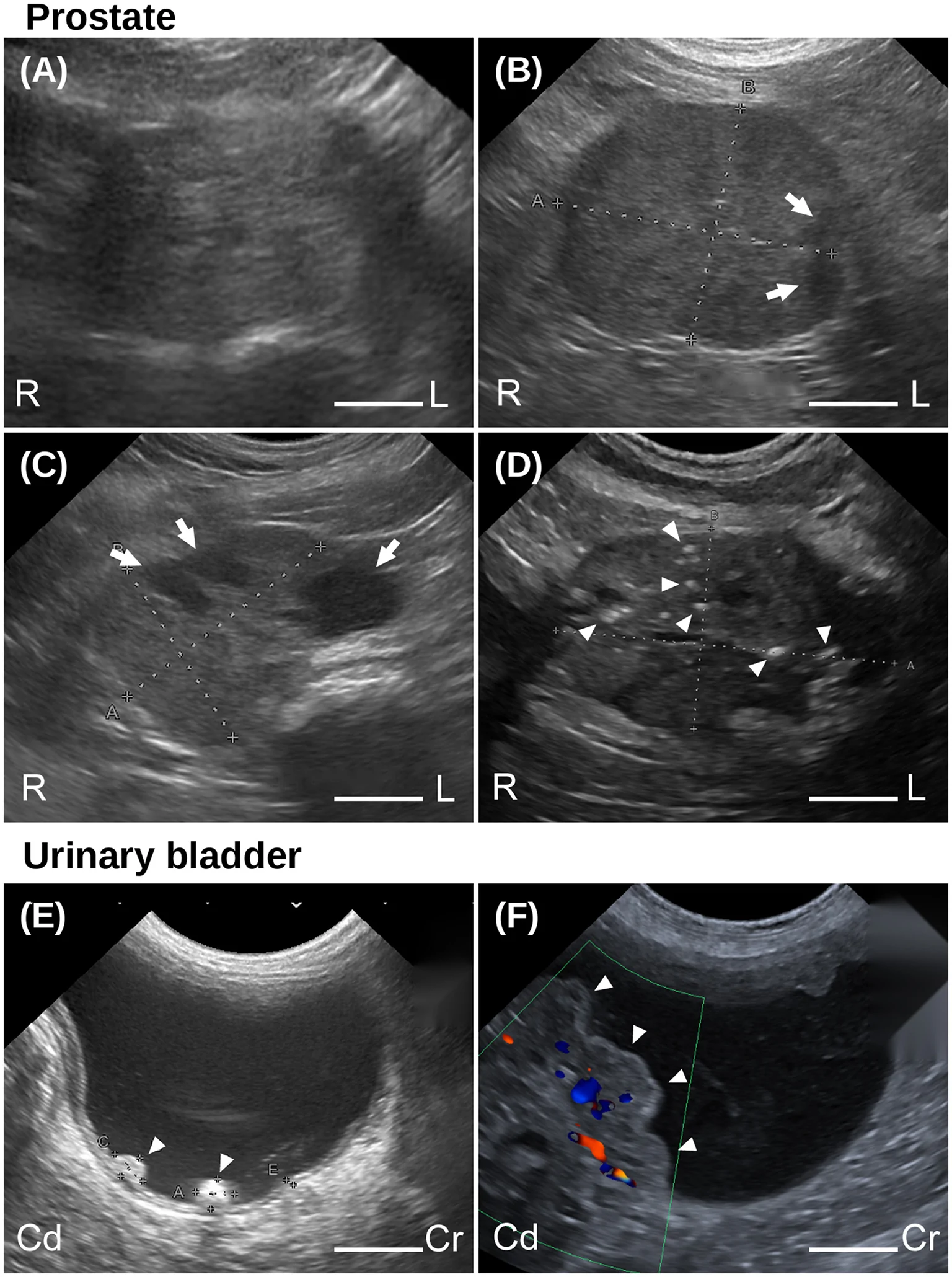
Time-course ultrasonography of the prostate and urinary bladder. **(A–D)** Prostate. R, right; L, left. **(A)** A normal ultrasonographic image of the prostate was obtained 3 years and 10 months before diagnosis, when the dog was intact. **(B)** On Day −62, heterogeneous echotexture and cystic hypoechoic lesions (arrows) were identified. **(C)** On Day 271, hypoechoic lesions (arrows) were observed in the prostatic parenchyma. **(D)** On Day 678, scattered hyperechoic areas (arrowheads) were observed throughout the prostate. **(E, F)** Urinary bladder. Cr, cranial; Cd, caudal. **(E)** On Day 565, multiple small masses (arrowheads) were detected in the bladder wall at the trigone. **(F)** On Day 852, a large mass lesion (arrowheads) was observed at the bladder trigone. Intralesional blood flow signals were detected within the lesion by color Doppler ultrasonography. A green outline indicates the color Doppler sampling box. All panels are shown at the same spatial scale. Scale bar = 1 cm. For display, the images were converted to grayscale [except **(F)**, which retains color Doppler information] and uniformly rescaled to a common spatial calibration.

### 2.1 Histopathological diagnosis

For differential diagnosis of the prostate lesion, we performed a Tru-cut biopsy (SuperCore™ Biopsy Instrument 18G, Argon Medical Device, TX, USA) of the prostate during cholecystectomy and submitted the sample for histopathological examination (Hist Vet, Kanagawa, Japan). Hematoxylin and eosin staining showed proliferation of epithelial cells forming irregular glandular lumens, cysts, and papillary structures. These neoplastic cells exhibited high-grade cellular atypia with abundant eosinophilic cytoplasm. Calcifications were also observed in some areas of the tissue. Based on these findings, the lesion was diagnosed as prostate carcinoma 15 days after the biopsy. We performed neither immunohistochemical staining to differentiate between prostatic adenocarcinoma and prostatic urothelial carcinoma (20, 21), nor a biopsy of the lung mass, as the pet owner did not want these additional examinations. We did not perform urine-based BRAF (V595E) mutation analysis (1) because the assay was not available at our institution at the time of diagnosis. We established the diagnosis of carcinoma histologically.

### 2.2 Treatment

On Day −43, we began treating the dog with meloxicam (Metacam^®^, Boehringer Ingelheim Vetmedica, Germany). On Day −1, however, imaging revealed prostate enlargement, and we assessed that disease control would be difficult with meloxicam alone. After discussing treatment options, the owner declined invasive procedures, including surgical removal of the prostate lesion and fine-needle aspiration of the lung mass. Systemic chemotherapy was reserved as an alternative if PAE proved technically infeasible. Thus, we performed PAE.

In PAE, we used tris-acryl gelatin microspheres (Embosphere^®^, Merit Medical Japan, Tokyo) as the embolic agent, with particle sizes ranging from 300 to 500 μm. PAE was performed under general anesthesia as follows. First, a 4Fr introducer sheath (Radifocus Introducer IIH, Terumo, Osaka) was inserted via the left carotid artery, and a 4Fr angiographic catheter (GraidCas II, straight, Terumo) was advanced into the left internal iliac artery under fluoroscopic guidance. Next, using a 2Fr microcatheter (Sniper 2, Terumo) and a microguidewire (Radifocus Guidewire M, Terumo), the catheter tip was positioned at the prostatic artery bifurcation beyond the vesical artery. An embolic agent diluted with contrast medium was injected slowly under fluoroscopic guidance, and the injection was stopped just before blood flow in the target vessel completely ceased. Lastly, the microcatheter was flushed with saline, and embolization was confirmed by angiography. The same procedure was performed on the right side. The total dose of the contrast medium was approximately 39.5 mgI/kg. The endovascular procedure took 52 min, and contrast-enhanced CT performed immediately after PAE, under the same anesthesia, served as the Day 0 imaging.

### 2.3 Time-course imaging

On Day 2, the dog was discharged without perioperative complications, except for mild hyperglycemia. For follow-up after PAE, we monitored the dog's general condition and performed whole-body contrast-enhanced CT scans under general anesthesia through Day 173 to evaluate lesions in the prostate and lungs. Thereafter, to avoid the burden of anesthesia, follow-up monitoring of the prostate was conducted by US, and that of lung lesions by thoracic radiography. For the prostate, the craniocaudal length (L), width (W), and depth (D; cm) were measured, and the volume (cm^3^) was calculated using the formula 1/2.6 × (L × W × D) + 1.8 (22). In addition, lung tumor volume (cm^3^) was calculated using the formula for an ellipsoid: 4/3 × π × L/2 × W/2 × D/2.

CT scans were performed on Days −62, 0, 33, and 173. On Day 33, multiple hypoattenuating lesions were observed within the prostatic parenchyma (Figure 1C). On Day 173, the hypoattenuating lesions enlarged, with marked gas accumulation (Figure 1D). Prostate volumes were as follows: Day −62, 11.55 cm^3^; Day 0, 13.82 cm^3^; Day 33, 11.51 cm^3^; and Day 173, 14.13 cm^3^ (Table 2). After PAE, the prostate volume decreased on Day 33 but increased with expansion of the hypoattenuating lesions on Day 173. To assess the remaining prostatic parenchymal volume, we calculated a corrected prostate volume by subtracting the volumes of hypoattenuation areas; this value was used only as a supportive reference. We found that the corrected prostate volumes on Days 33 and 173 were 9.29 cm^3^ and 10.16 cm^3^, respectively, both lower than the Day 0 volume (13.82 cm^3^; Table 2). Later, prostate volume was measured by US and remained 10–11 cm^3^ until Day 481. From Day 678, the prostate volumes increased, reaching 16.42 cm^3^ and 16.56 cm^3^ on Days 804 and 852, respectively (Table 2).

**Table 2:** Time-course changes in prostatic and pulmonary mass volumes measured by CT and US, and thoracic radiography before and after prostatic artery embolization (PAE).

Prostatic volume (cm^3^)												
Measurement	Day											
	−62	0	33	173	271	314	398	481	678	712	804	852
CT volume	11.55	13.82	11.51	14.13	—	—	—	—	—	—	—	—
Corrected CT volume^*^	—	—	9.29	10.16	—	—	—	—	—	—	—	—
US volume	9.52	—	—	—	10.18	10.01	10.16	11.04	13.10	12.97	16.42	16.56
Pulmonary mass volume (cm^3^)												
Measurement	Day											
	−62	0	33	173	405	712	740	768	804	824	852	889
CT volume	0.28	0.47	0.52	0.89	—	—	—	—	—	—	—	—
Radiographic volume	0.65	0.89	—	1.15	1.84	4.84	4.97	6.34	7.95	9.99	12.20	14.40

Day 0 was defined as the day of PAE; the Day 0 CT was obtained immediately after PAE. ^*^Corrected CT volume was calculated after excluding hypoattenuating lesions identified on CT. Pulmonary mass volumes were calculated from both CT and thoracic radiographs through Day 173 to assess agreement between the two imaging modalities; thereafter, pulmonary lesions were monitored by thoracic radiographs alone to avoid repeated general anesthesia.

—, not evaluated at that time point.

CT, computed tomography; PAE, prostatic artery embolization; US, ultrasonography.

After Day 271, the prostate and bladder were monitored by US. The bladder was monitored to assess whether prostate carcinoma had spread to the bladder wall. On Day 271, multiple hypoechoic lesions were observed within the prostatic parenchyma (Figure 2C). On Day 678, hyperechoic lesions were observed throughout the prostate (Figure 2D). On Day 565, multiple small masses were observed within the bladder wall at the trigone (Figure 2E). On Day 852, these masses had fused into a large mass, and abundant blood flow signals were detected within the lesion (Figure 2F).

We monitored the lung lesions by contrast-enhanced CT scans and thoracic radiographs until Day 173 and observed a solitary, oval-shaped mass. We estimated the lung tumor volume using CT images or two-view thoracic radiographs (Table 2). Although the lung tumor volumes estimated from the two-view thoracic radiographs were larger than those from the CT images, both methods showed a consistent trend of gradual increase over time; thus, follow-up of the lung lesion after Day 173 was performed using thoracic radiographs. The lung tumor volumes estimated from thoracic radiographs were 0.89 cm^3^ on Day 0 and 1.84 cm^3^ on Day 405, indicating a relatively slow increase. It then increased rapidly to 4.84 cm^3^ on Day 712 and 14.40 cm^3^ on Day 889 (Table 2). In addition, enlargement of the medial iliac lymph node was observed on Day 712, and it had become a distinct mass on Day 804.

Urinary signs were observed temporarily on two occasions: urinary leakage on Day 173, and pollakiuria with stranguria accompanied by fever on Day 438. Urine cultures were positive for bacteria on both occasions. Except for these episodes, a good general condition was maintained until Day 740; we did not observe clinical signs related to defecation disorders throughout the disease course. We continued meloxicam after PAE and switched to robenacoxib on Day 426 due to a progressive increase in blood urea nitrogen; we discontinued NSAID therapy on Day 893 because of deterioration in the dog's general condition. Beginning on Day 768, the dog developed recurrent appetite loss and lethargy and died on Day 918 after developing cachexia and respiratory failure.

## 3 Discussion

Previously, Culp et al. (18) reported that PAE for canine prostatic carcinoma improved clinical signs and reduced prostate volume, with imaging evaluations of prostate volume reduction conducted up to 30 days after PAE; the subsequent time course of prostatic changes remains unclear. In the current case report, we monitored imaging progression of the prostate lesions for approximately 900 days following PAE.

In dogs, prostatic imaging after PAE has been reported in BPH; magnetic resonance imaging (MRI) and US showed prostatic infarcts and cavitation early after PAE (12–15). Consistent with imaging studies, histological changes in the prostate after PAE have also been reported in BPH, including cavitation, glandular atrophy, and stromal fibrosis, which may be due to ischemia, infarction, and necrosis (12–14). Similar imaging and histological changes have been reported in human BPH cases (9), in which infarcts were identified as non-enhancing areas on contrast-enhanced MRI (23). In our case, we observed hypoattenuating lesions in the prostate on contrast-enhanced CT on Day 33, which enlarged on Day 173. On Day 271, US showed multiple hypoechoic lesions in the prostate. These findings were consistent with ischemia, infarction, and cavitation reported in canine BPH cases after PAE (12–15), suggesting that ischemic changes were also induced in the prostate in our case.

Unlike the hypoattenuating and hypoechoic lesions observed early post-PAE, we observed scattered hyperechoic lesions on Day 678, which have not been reported previously; this finding may represent a unique change emerging during the chronic phase. In dogs, 1 month after PAE, cavitation and infarction of the prostate have been reported in BPH using US (Sun et al.) and MRI (Lucas-Cava et al.), respectively (13, 24), with no long-term imaging follow-up of these lesions. Three months after PAE, Jeon et al. reported glandular atrophy and stromal fibrosis of the prostate on MRI, with histological findings showing residual normal glandular tissue in the prostate (12).

In human biopsy samples, Camara-Lopes (9) and Mordasini (10) reported necrosis and fibrotic changes at 6 weeks post-PAE, although they examined only a single time point. In human BPH, time-course MRI over 12–18 months monitored PAE-induced prostatic infarcts, with central-gland infarcts progressively decreasing in size and becoming isointense to the surrounding parenchyma (23). The scattered hyperechoic lesions observed in our case on Day 678 (approximately 22 months after PAE) may have reflected chronic-phase tissue remodeling, a phenomenon not previously reported.

We also observed a reduction in prostatic parenchymal volume. Although this corrected CT volume has not been validated as a quantitative measure of true prostatic parenchymal volume and depends on observer delineation of the hypoattenuating lesions, excluding these lesions allowed a descriptive assessment of the remaining enhancing tissue. Lucas-Cava et al. (24) reported a correlation between the extent of infarction after PAE and prostate volume reduction, suggesting that the absorption of infarcted tissue may contribute to prostate shrinkage. Similarly, in our case, although an increase in prostate volume was observed on Day 173 due to expansion of the hypoattenuating lesions, the corrected prostate volume—calculated by excluding these lesions—had decreased. Culp et al. (18) reported that prostate volume decreased by a median of 39.4% on CT approximately 30 days after PAE in 20 dogs with prostatic carcinoma. In our case, the corrected prostate volume decreased by approximately 33%, closer to the finding of Culp et al.; the volume reduction on Day 33 without correction was approximately 17%. In human BPH, a significant reduction in prostate volume after PAE (an average of 32.0% over 12–18 months) was also observed in cases with prostatic infarcts (23). These findings suggest that absorption of infarcted tissue and a reduction in prostatic parenchymal volume led to an overall decrease in prostate volume. Although cavitation could contribute to an increase in prostate volume, infarcts and necrotic tissue appeared to be resorbed, reducing prostatic parenchymal volume. We also observed that the prostate volume calculated by US showed no clear regrowth until Day 481, demonstrating that the reduction in prostatic parenchymal volume after PAE was maintained for a long period.

Prostatic carcinoma in dogs is highly locally invasive, and at the time of diagnosis, cases often involve invasion of the bladder neck or prostatic urethra (3, 4). In our case, we observed local invasion into the bladder trigone and tumor formation in the bladder wall only during the later stages of the disease. We observed a distinct intramural mass on Day 565, with slow progression of the local lesion thereafter. Since the corrected prostate volume decreased after PAE and no increase was observed until Day 481, PAE may affect not only tumor growth but also local invasion over a long period. This assumption needs to be clarified by future accumulation of prostatic cancer cases after PAE.

The current case involved a lung lesion that grew slowly through Day 405; enlargement and swelling of the medial lymph node were detected from Day 712. Thus, the slow progression of both the prostate and lung lesions over a long period appeared to contribute to the favorable long-term outcome in this case, although no histological examination was performed to precisely characterize the lesions.

The dog had no recorded history of prostatitis, prostatic cysts, or hyperplasia, and prostatic ultrasonography performed 3 years and 10 months before diagnosis was normal. Thus, the current case did not have pre-existing prostatic disease that could predispose the dog to subsequent prostatic neoplasia.

On Days 173 and 438, the dog temporarily exhibited urinary signs (urinary incontinence and pollakiuria, respectively), and urine cultures showed bacterial infection. Since these signs improved with antibiotic therapy, bacterial prostatitis or a urinary tract infection could cause the clinical signs (1, 3). On Day 173, we observed a gas-filled area in the prostate, which we associated with ischemic and necrotic changes after PAE. The area could also be associated with bacterial infection, since *K. pneumoniae*, a gas-producing bacterium (25), was isolated from a urine culture at the time point. Although serious complications such as persistent urinary incontinence, bladder wall necrosis, and peritonitis have been reported after PAE (8, 26), the urinary signs in our case were transient and resolved with antibiotic therapy, and no serious PAE-related complications were observed.

One limitation of the current case report was that we did not assess oxidative stress or redox biomarkers. Although oxidative stress has been implicated in canine BPH (2), the potential relationship between post-PAE redox homeostasis and long-term outcome could not be evaluated in this case.

## 4 Conclusion

We reported time-course imaging over approximately 900 days after PAE in a case of canine prostatic carcinoma with long-term survival. Using CT and US, we observed that the prostatic lesions shifted from hypoattenuating and hypoechoic to scattered hyperechoic areas, with a reduced prostatic parenchymal volume. Although local cancer invasion was observed, clinical disease progression was slow. In summary, this report provides novel longitudinal imaging data over approximately 900 days after PAE and supports further evaluation of PAE as a minimally invasive treatment option for canine prostatic carcinoma.

## Patient perspective

Because this is a veterinary case report, the patient's perspective does not apply. The owner consented to the medical treatment and to the publication of this report.

## Data Availability

The original contributions presented in the study are included in the article/Supplementary material, further inquiries can be directed to the corresponding authors.
