# Supplementary material for: Long-term CT and US imaging of canine prostatic carcinoma after prostatic artery embolization: a 900-day follow-up case report
Source: Front Vet Sci. 2026 Sep 28;13:1947473. doi: 10.3389/fvets.2026.1947473 (PMC13656744; doi:10.3389/fvets.2026.1947473)
Supplement: Supplementary file 1 [file Table_1.docx]

**Supplementary Material — CARE Checklist (2013)**

Manuscript: Long-term CT and US imaging of canine prostatic carcinoma after prostatic artery embolization: a 900-day follow-up case report.

This case report was prepared and reported in accordance with the CARE (CAse REport) guidelines (2013). The table below maps each CARE checklist item to the corresponding section and page(s) of the submitted manuscript.

*This is a veterinary case report. Human-specific CARE items are adapted as follows: “patient” refers to the animal; “patient perspective” (item 12) is not applicable; and “informed consent” (item 13) refers to written consent obtained from the owner. Page numbers refer to the revised manuscript.*

| **Topic** | **Item** | **CARE checklist item** | **Reported in manuscript (section / page)** |
| --- | --- | --- | --- |
| **Title** | 1 | Includes the area of focus (diagnosis and intervention) and the words “case report.” | Title, p.1: “Long-term CT and US imaging of canine prostatic carcinoma after prostatic artery embolization: a 900-day follow-up case report.” |
| **Key words** | 2 | 2–5 key words identifying diagnoses/interventions, including “case report.” | Keywords, p.1.  *Note: “case report” is not listed as a keyword; it appears in the title. Add “Case Report” as a keyword if strict CARE wording is preferred.* |
| **Abstract** | 3a | Introduction — what is unique and what it adds to the literature. | Abstract, p.1: long-term intraprostatic imaging after PAE is poorly documented; the longest time-course imaging reported after PAE in a dog. |
|  | 3b | Main symptoms and/or important clinical findings. | Abstract, p.1 (clinical signs well controlled; imaging findings). *Fuller presenting signs are in Case Description, p.2.* |
|  | 3c | Main diagnoses, therapeutic interventions, and outcomes. | Abstract, p.1: prostatic carcinoma; PAE; ~900-day imaging course; survival to Day 918. |
|  | 3d | Conclusion — main take-away lesson(s). | Abstract, p.1: PAE as a feasible, minimally invasive option; sustained local disease control. |
| **Introduction** | 4 | Brief background summary referencing the relevant literature. | Introduction, pp.1–2 (refs 1–19). |
| **Patient information** | 5a | De-identified patient-specific information. | Case Description, p.2: 13-year-6-month-old neutered male Chihuahua, 7.76 kg. |
|  | 5b | Primary concerns and symptoms of the patient. | Case Description, p.2: acute vomiting, anorexia, and polydipsia; presented on the day the owner first noticed the signs; no urinary or respiratory signs were noted, with reportedly normal urination and defecation. |
|  | 5c | Relevant medical / (family, psychosocial) history. | Case Description, p.2: well-controlled diabetes mellitus diagnosed 4 years earlier, managed with twice-daily insulin; castration at 11 years of age concurrently with excision of perianal gland adenoma. Family/psychosocial history not applicable (veterinary case). |
|  | 5d | Relevant past interventions and their outcomes. | Case Description / Treatment, pp.2–3: cholecystectomy for gallbladder lesion; meloxicam from Day −43. |
| **Clinical findings** | 6 | Relevant physical examination and other clinical findings. | Case Description, pp.2–3: baseline blood examination (elevated hepatobiliary enzymes, hyperbilirubinemia, hyperlactatemia, mild thrombocytopenia, hyperglycemia); prostatic enlargement with heterogeneous enhancement (CT); heterogeneous echotexture and cystic hypoechoic areas (US); solitary mass in the left caudal lung lobe (CT/thoracic radiography). |
| **Timeline** | 7 | Important dates and times, as a table or figure. | Figure 1A (timeline) and dated narrative in Case Description / Time-course imaging, pp.2–4; PAE = Day 0; Table 2 (dated prostatic and pulmonary volumes), pp.11–12. Figure 1 legend, p.9. |
| **Diagnostic assessment** | 8a | Diagnostic methods (examination, laboratory, imaging). | Case Description / Histopathological diagnosis, pp.2–3: baseline hematology and biochemistry, contrast-enhanced CT, US, thoracic radiography, Tru-cut biopsy with H&E (diagnosis established 15 days after biopsy), urine culture. |
|  | 8b | Diagnostic challenges (access, financial, cultural). | Histopathological diagnosis, p.3: the owner declined immunohistochemical subtyping and lung-mass biopsy; urine-based BRAF (V595E) analysis was not performed (assay not available at the time of diagnosis). |
|  | 8c | Diagnostic reasoning, including other diagnoses considered. | Histopathological diagnosis, p.3: carcinoma on H&E; adenocarcinoma vs. urothelial carcinoma not differentiated (refs 20, 21). |
|  | 8d | Prognostic characteristics (e.g., staging) where applicable. | Introduction, p.1 (prognosis of canine prostatic carcinoma); Time-course imaging, p.4 (suspected pulmonary lesion, medial iliac lymph-node enlargement). |
| **Therapeutic intervention** | 9a | Types of intervention (pharmacologic, surgical, etc.). | Treatment, p.3: prostatic artery embolization (PAE); meloxicam (later robenacoxib); antibiotic therapy (Time-course imaging, p.4). |
|  | 9b | Administration (dosage, strength, duration). | Treatment, p.3: tris-acryl gelatin microspheres (Embosphere, 300–500 µm); catheter/microcatheter details; contrast dose ~39.5 mgI/kg; procedure time 52 min; NSAID durations (meloxicam Day −43 to 426; robenacoxib Day 426 to 893), Time-course imaging, p.4. |
|  | 9c | Changes in intervention, with rationale. | Treatment / Time-course imaging, pp.3–4: meloxicam (Day −43) escalated to PAE (Day 0) as control was insufficient; meloxicam continued after PAE and switched to robenacoxib on Day 426 (progressive BUN increase), with NSAID therapy discontinued on Day 893; antibiotic therapy on Days 173/438. |
| **Follow-up and outcomes** | 10a | Clinician- and patient-assessed outcomes. | Time-course imaging, p.4: a good general condition was maintained until Day 740; survival to Day 918. |
|  | 10b | Important follow-up diagnostic and test results. | Time-course imaging, p.4; Table 2 (longitudinal prostatic and pulmonary volumes), pp.11–12; Figure 1B–D (time-course CT) and Figure 2 (time-course US) over ~900 days; figure legends, pp.9–10. |
|  | 10c | Intervention adherence and tolerability. | Time-course imaging, p.4: discharged Day 2 without perioperative complications except mild hyperglycemia; NSAID switched to robenacoxib (Day 426) due to a progressive BUN increase and discontinued on Day 893. |
|  | 10d | Adverse and unanticipated events. | Time-course imaging / Discussion, p.4 and p.6: transient urinary signs (Days 173 and 438) with bacterial infection, responsive to antibiotics; BUN increase leading to NSAID switch; no serious PAE-related complications. |
| **Discussion** | 11a | Strengths AND limitations of the case report. | **Strength:** to our knowledge, this is the longest post-PAE time-course imaging record in a dog with prostatic carcinoma; imaging progression was monitored for approximately 900 days (Abstract; Discussion, opening paragraph; chronic-phase remodeling described as "not previously reported").  **Limitations are reported within the Case Description and Discussion narrative rather than as a separate paragraph: (1) single case — the need for further cases is stated ("This assumption needs to be clarified by future accumulation of prostatic cancer cases after PAE," Discussion); (2) the pulmonary lesion was not histologically confirmed (lung-mass biopsy declined by the owner, Histopathological diagnosis; "no histological examination was performed to precisely characterize the lesions," Discussion); (3) immunohistochemical subtyping was not performed ("We performed neither immunohistochemical staining to differentiate between prostatic adenocarcinoma and prostatic urothelial carcinoma," Histopathological diagnosis); (4) the corrected prostate volume is an exploratory, non-standardized measure used only as a supportive reference (Time-course imaging; Discussion; Table 2 footnote). (5) oxidative stress and redox biomarkers were not evaluated (Discussion, final paragraph, p.6); (6) because no pre-existing prostatic disease was documented, a possible predisposing role of prior prostatic disease could not be assessed (Discussion, p.6).** |
|  | 11b | Discussion of the relevant literature with references. | Discussion, pp.4–6 (refs 1–4, 8–10, 12–15, 18, 23–26). |
|  | 11c | Scientific rationale for conclusions (incl. possible causes). | Discussion, pp.4–6: ischemic/cavitary/chronic-remodeling changes and parenchymal-volume reduction as basis for local control. |
|  | 11d | Primary take-away lesson(s) in a one-paragraph conclusion (no references). | Conclusion, p.6. |
| **Patient perspective** | 12 | The patient shares their perspective where appropriate. | Patient Perspective, p.6: not applicable (veterinary case); the owner consented to treatment and publication. |
| **Informed consent** | 13 | Did the patient give informed consent? | Ethics Statement, p.7: Yes — written informed consent obtained from the owner for treatment and publication. |

*Reference: Gagnier JJ, Kienle G, Altman DG, Moher D, Sox H, Riley D; CARE Group. The CARE guidelines: consensus-based clinical case reporting guideline development. 2013.*
